# Anabolic Implants Varying in Hormone Type and Concentration Influence Performance, Feeding Behavior, Carcass Characteristics, Plasma Trace Mineral Concentrations, and Liver Trace Mineral Concentrations of Angus Sired Steers

**DOI:** 10.3390/ani11071964

**Published:** 2021-06-30

**Authors:** Caleb C. Reichhardt, Elizabeth M. Messersmith, Tevan J. Brady, Laura A. Motsinger, Reganne K. Briggs, Brett R. Bowman, Stephanie L. Hansen, Kara J. Thornton

**Affiliations:** 1 Department of Animal, Dairy and Veterinary Science, Utah State University, Logan, UT 84322, USA; ccreichhardt@gmail.com (C.C.R.); tjamesbrady@gmail.com (T.J.B.); laura_smith95@live.com (L.A.M.); reganne.briggs@gmail.com (R.K.B.); brett.bowman@usu.edu (B.R.B.); 2 Department of Animal Science, Iowa State University, Ames, IA 50011, USA; emm2@iastate.edu

**Keywords:** estradiol, growth, implants, manganese, trenbolone acetate, zinc

## Abstract

**Simple Summary:**

Though anabolic implants are commonly utilized in U.S. cattle production, comparisons between hormone type and content of different implants and the effects on growth and trace mineral stores is limited. The objective of this study was to evaluate the effects of anabolic implants varying in hormone type and concentration on growth, carcass characteristics, and trace mineral concentrations in Angus steers. Cattle administered an estradiol only implant did not experience differences in growth compared to non-implanted controls. However, cattle implanted with a trenbolone acetate only implant or a combined (estradiol and trenbolone acetate) implant experienced improvements in growth and changes in plasma and liver trace mineral concentrations. Greatest differences in growth and trace mineral concentrations were observed in steers administered the combination implant compared to non-implanted controls. These data suggest hormone type and concentration influence implant-induced growth and changes in plasma and liver trace mineral concentrations.

**Abstract:**

Fifty Angus-sired steers were utilized to evaluate the effects of anabolic implants varying in hormone type and concentration on performance, carcass traits, and plasma and liver trace mineral concentrations over 129 d. Steers were stratified by weight into one of four (*n* = 12 or 13/treatment) implant treatments: (1) estradiol (E2; 25.7 mg E2; Compudose, Elanco Animal Health, Greenfield, IN, USA), (2) trenbolone acetate (TBA; 200 mg TBA; Finaplix-H, Merck Animal Health, Madison, NJ, USA), (3) combination implant (ETBA; 120 mg TBA + 24 mg E2; Revalor-S, Merck Animal Health), or (4) no implant (CON). Steers were randomly assigned to pens equipped with GrowSafe bunks and fed a corn and barley-based finishing ration. Overall average daily gain and body weight were greater for ETBA and TBA than CON (*p* ≤ 0.04), but not E2 (*p* ≥ 0.12). Feed efficiency and hot carcass weight were only greater than CON for ETBA (*p* ≤ 0.03). Plasma and d 2 liver Zn concentrations were lesser for ETBA than CON (*p* ≤ 0.01) and d 10 liver Mn was lesser (*p* = 0.0003) for TBA than CON. These data indicate that implants containing TBA influence growth and trace mineral parameters, though more work investigating this relationship is necessary.

## 1. Introduction

As the global population increases [1], beef production is faced with challenges related to the changing climate and use of limited resources [2,3]. It is imperative that efficiency of cattle production is improved to increase both environmental and economic sustainability aspects of beef production. One method to improve sustainability is through the use of anabolic implants. Anabolic implants decrease greenhouse gas emissions by 8.9%, and overall land use by 9.1% [2], effectively decreasing the environmental impact of beef production.

Anabolic implants contain steroid hormones to increase the efficiency and growth rate of cattle [4,5,6], and have been routinely used in cattle production since the late 1950s in the U.S. [7]. Although not all countries allow the use of anabolic implants in cattle production, there are currently over 40 commercially available implants approved by the Food and Drug Administration in the United States. These anabolic implants are approved for all stages of beef production; from suckling calves to finishing cattle [5]. Roughly 90% of all cattle on feed in the U.S. receive at least one anabolic implant during production, with 80% receiving two or more [8]. Anabolic implants can typically be classified as estrogenic, typically containing estradiol (E2), androgenic, typically containing the synthetic testosterone analogue trenbolone acetate (TBA), or as combined, being composed of both estrogenic and androgenic hormones [7].

Anabolic implants increase economically viable traits such as average daily gain (ADG), feed intake, feed efficiency (FE), hot carcass weight (HCW), and ribeye area (REA) [4,7]. However, the exact physiological and molecular mechanisms by which anabolic implants operate to increase skeletal muscle growth in cattle remains elusive [9,10,11,12]. Furthermore, the increase in growth caused by anabolic implants, may increase trace mineral requirements to support skeletal muscle growth [13], as lambs implanted with zeranol tended to retain greater amounts of Zn, and lose less Cu and Mn in feces and urine [14]. When trace minerals are supplemented above national research council recommendations at feedlot consultant recommended concentrations, cattle receiving anabolic implants increase growth even further [13]. Our hypothesis is that varying hormone type and concentration will alter economically viable traits and mineral status of Angus sired steers. Due to the complex nature of anabolic implants, and the unknowns in their mechanisms of operation to increase skeletal muscle growth, the purpose of this research was to investigate feedlot performance of steers receiving an estradiol only implant (E2), a trenbolone acetate only implant (TBA), or a combined estradiol and trenbolone acetate implant (ETBA) compared to non-implanted steers (CON). Additionally, due to the importance of trace minerals in growth, liver and plasma mineral concentrations were evaluated to better understand the relationship between anabolic implants and trace minerals in beef cattle. 

## 2. Materials and Methods

All live animal procedures and protocols for this experiment were approved by the Utah State University Institutional Animal Care and Use Committee (IACUC Protocol #2817). 

### 2.1. Animals, Experimental Design and Treatments

This experiment was conducted at the Utah State University feedlot and was run concurrently with a previously published study [15]. As such, the animals in this study were treated similarly to the previously published study [15]. Fifty Angus sired steers (327 kg ± 25 kg) out of commercial Angus cows that had not received any prior growth promotant treatments were stratified by weight at the start of the trial. Prior to beginning the trial each steer received an electronic (EID) and visual ear tag. Steers were assigned to one of four implant treatments: (1) estradiol only implant containing 25.7 mg estradiol (E2; *n* = 12; Compudose, Elanco Animal Health, Greenfield, IN, USA), (2) trenbolone acetate only implant containing 200 mg trenbolone acetate (TBA; *n* = 12; Finaplix-H, Merck Animal Health, Madison, NJ, USA), (3) a combined implant containing 120 mg trenbolone acetate and 24 mg estradiol (ETBA; *n* = 13, Revalor-S, Merck Animal Health), or (4) no implant (CON; *n* = 13). Steers were randomly placed into one of four covered pens equipped with two GrowSafe bunks (GrowSafe Systems Ltd.; Airdrie, AB, Canada) per pen to measure individual feed disappearance via radio frequency EID tags. Steers utilized in this trial were housed with other steers of similar size from the Utah State University beef herd (*n* = 15 steers/pen). Steers underwent a two week adaptation period to the system prior to beginning the trial. Steers always had free choice access to water and were fed the same diet. Diets were stepped up between 10 and 12% (DM basis) concentrate every 10 d from a backgrounding ration consisting of 40% (DM basis) concentrate to a finishing ration consisting of 86% (DM basis) concentrate (Table 1) over a 41 day period after implanting and the start of the trial. Three animals were removed from the trial due to bloat (*n* = 1, E2), an abdominal abscess (*n* = 1, ETBA), and a hock injury (*n* = 1, ETBA). These were not related to their respective treatments, but prompted the removal of all 3 animals from all analyses, except the liver trace mineral analyses and the serum trace mineral analyses as animal removal occurred after day 30 of the trial. 

### 2.2. Feedlot Performance and Sample Collection

Individual as-fed feed intake was measured by the GrowSafe system. A minimum of three feed samples per ration were collected and analyzed at a commercial lab (Cumberland Valley Analytical Services, Waynesboro, PA, USA). Daily feed intake was converted to dry matter intake (DMI) by utilizing as-fed feed intake and the percent DM of each ration. Steers were weighed individually on a certified scale (Tru-Trust GR3000, College Station, Texas) and ultrasound was conducted by a certified ultrasound technician using a portable ExaGo ultrasound (Universal Imaging, Bedfords Hills, NY, USA) on days 0, 28, 56, 84, and 112. Weights, ADG, and 12th rib fat thickness were recorded. Individual ADG was calculated by subtracting the initial body weight (BW) for the period from the final BW for the period and dividing by the number of days for that period. Gain to feed (G:F) for individual steers was calculated by dividing ADG by DMI for each period. Blood was collected and harvested as serum via jugular puncture on days 0, 2, 10, 28, 56, 84, 112, and 129 using 10.0 mL, 16 × 100 mm BD vacutainer serum blood collection tubes. Blood was collected and harvested as plasma via jugular puncture on days 0, 2, 10, and 30 using 6 mL,13 ×100 mm BD vacutainer plasma blood collection tubes containing trace mineral grade K_2_EDTA. Blood samples were allowed to coagulate and kept on ice and transported approximately 12 km to the laboratory. Blood samples were centrifuged at 1000× *g* for 15 min at 4 °C. Supernatants were then collected, aliquoted, and blood samples were stored at −20 °C until further analysis. Liver biopsies were performed on days 2 and 10 post-implanting. Liver samples were collected with a liver biopsy kit performed by Utah State University’s clinical veterinarians using the TruCut method [16]. Liver was extracted between the 11th and 12th rib space on the right-hand side of the steer. Plasma and liver samples were collected from all steers, but only 12 steers/treatment were analyzed to ensure equal numbers across treatments.

### 2.3. Feeding Behavior Data

All feeding behavior data were analyzed based off the two main categorical traits calculated by the GrowSafe bunks; (1) bunk visit (BV), which is the single reading of an EID tag when entering a bunk, whether it consumed feed or not, and (2) feed bouts (FB), which is the reading of a single animal EID tag when entering a bunk, and a minimum of 10 g of feed were consumed, and following previously published methodology [17]. Based off BV, the average duration of the BV (ABVD), the average amount of feed consumed per BV (ABVC), and the amount of time an animal spent with its head down per BV (ABVHD) were analyzed. Regarding FB data, the following was also calculated: the duration of the FB (DFB), the average amount of feed consumed per FB (AFFB), and the average time an animal’s head was down while it consumed feed during a BV (HDFB). 

### 2.4. Trace Mineral Analysis

Plasma and liver samples were shipped overnight on dry ice to Iowa State University and stored at −20 °C until analysis. Trace mineral analysis of plasma samples was conducted using inductively coupled plasma optical emission spectrometry (Optima 7000 DV, Perkin Elmer, Waltham, MA, USA) via previously described methods [18]. Liver samples were analyzed for Cu, Fe, Mn, and Zn via inductively coupled plasma mass spectroscopy (Analytik Jena Inc., Jena, Thuringia, Germany) at the Iowa State University Veterinary Diagnostic Laboratory. To ensure instrument accuracy, a quality control standard for plasma (Trace Elements Serum Control #66816; UTAK Laboratories Inc., Valencia, CA, USA) and liver (Bovine Liver #1577c; National Institute of Standards and Technology, Gaithersburg, MD, USA) analysis was utilized on each run. 

### 2.5. Serum Urea Nitrogen

A commercially available colorimetric assay was used to detect serum urea nitrogen (SUN) in duplicate (Invitrogen, Urea Nitrogen BUN Colorimetric Detection Kit; ThermoFisher Scientific, Waltham, MA, USA). The plate was read on a BioTek all-in-one microplate reader using Gen5d 2.0 all-in-one microplate reader software (BioTek Instruments, Winooski, VT, USA). Intra-assay CV: 1.94%. Inter-assay CV: 2.41%.

### 2.6. Carcass Characteristics 

Steers were shipped at an average of 7 mm 12th rib fat. This trial occurred in May 2020 during the COVID-19 pandemic; therefore, steers were shipped early to ensure they could be harvested. The steers were harvested at a commercial facility (Hyrum, UT, USA). Dressing percentage, HCW, marbling score, REA, ribeye fat thickness, and cold camera yield grade were recorded at the plant by trained USDA inspectors. Dressing percentage was calculated by dividing HCW by final live weight with a 4% shrink and multiplying by 100. 

Carcass adjusted final BW was calculated by dividing the HCW by the individual steer’s dressing percentage. To assess overall carcass adjusted gain, initial BW with a 4% shrink was subtracted from the carcass adjusted final BW. Overall carcass adjusted gain was divided by the total number of days on feed to determine carcass adjusted ADG and carcass adjusted G:F was calculated by dividing the total carcass-adjusted gain by the individual steer’s total DMI.

### 2.7. Statistical Analysis

Fifty Angus steers were initially stratified by weight and assigned to one of four treatments. Statistical analysis was performed using the MIXED procedure of SAS (version 9.4; SAS Inst. Inc., Cary, NC, USA) with the fixed effect of treatment. Contrast statements were constructed to test each treatment vs. CON (E2 vs. CON, TBA v. CON, and ETBA vs. CON). Furthermore, BW, DMI, G:F, feeding behavior, plasma trace mineral, and SUN data were analyzed as repeated measures with the repeated effect of time and day 0 values used as a covariate in analysis. Data were tested for outliers using Cook’s D statistical test. All data are presented as the least square mean ± SEM. Statistical significance was determined at *p* ≤ 0.05 and tendency at 0.05 < *p* ≤ 0.10. 

## 3. Results

### 3.1. Live Animal Performance

**Figure 1:**
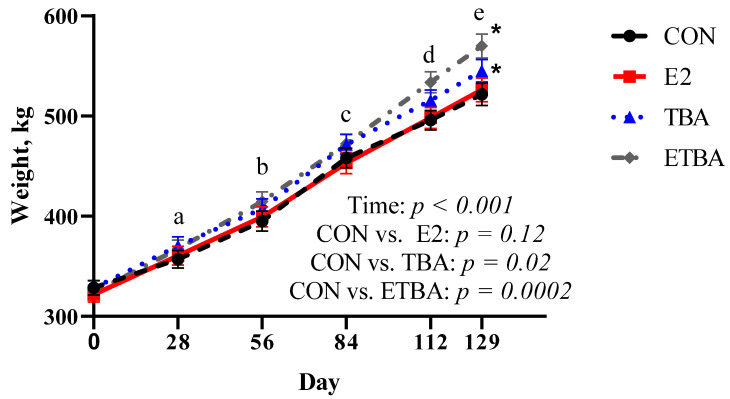
Weights were measured as described in the Materials and Methods. Steers were initially stratified by weight and assigned to one of four treatments: (1) estradiol (E2) only implant containing 25.7 mg E2 (*n* = 12; Compudose, Elanco Animal Health), (2) trenbolone acetate (TBA) only implant containing 200 mg TBA (*n* = 12; Finaplix-H, Merck Animal Health), (3) a combined estradiol and trenbolone acetate (ETBA) implant containing 120 mg TBA and 24 mg E2 (*n* = 13, Revalor-S, Merck Animal Health), or (4) no implant (CON; *n* = 13). Different letters indicate a difference (*p* ≤ 0.05) in weights between the time points. Contrasts were used to compare differences in weight gain over time between treatment and the control. The *p*-values for this analysis are displayed on the figure. The * indicates a difference (*p* < 0.05) between that treatment and the CON steers when analyzed as a repeated measure over time. All data are reported as LSMEANS ± SEM.

**Figure 2:**
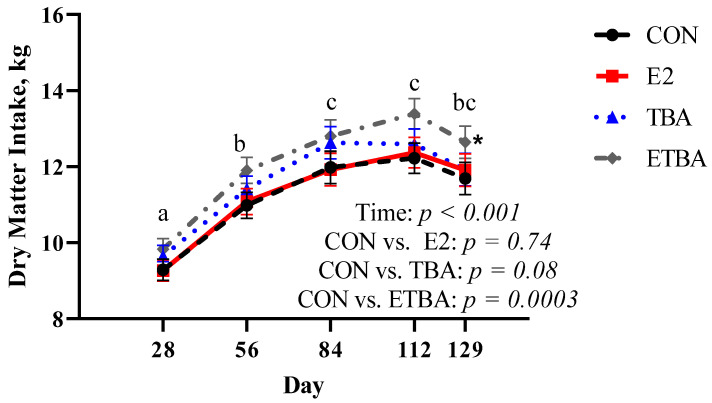
Dry matter intake was measured as described in the Materials and Methods. Steers were initially stratified by weight and assigned to one of four treatments: (1) estradiol (E2) only implant containing 25.7 mg E2 (*n* = 12; Compudose, Elanco Animal Health), (2) trenbolone acetate (TBA) only implant containing 200 mg TBA (*n* = 12; Finaplix-H, Merck Animal Health), (3) a combined estradiol and trenbolone acetate (ETBA) implant containing 120 mg TBA and 24 mg E2 (*n* = 13, Revalor-S, Merck Animal Health), or (4) no implant (CON; *n* = 13). Different letters indicate a difference (*p* ≤ 0.05) between time points. Contrasts were used to compare differences in dry matter intake over time between treatment and the control. The *p*-values for this analysis are displayed on the figure. The * indicates a difference (*p* < 0.05) between that treatment and the CON steers when analyzed as a repeated measure over time. All data are reported as LSMEANS ± SEM.

**Figure 3:**
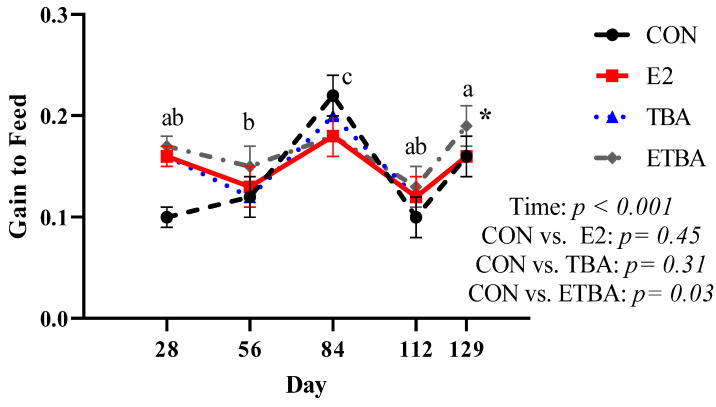
Gain to Feed (G:F) was measured as described in the Materials and Methods. Steers were initially stratified by weight and assigned to one of four treatments: (1) estradiol (E2) only implant containing 25.7 mg E2 (*n* = 12; Compudose, Elanco Animal Health), (2) trenbolone acetate (TBA) only implant containing 200 mg TBA (*n* = 12; Finaplix-H, Merck Animal Health), (3) a combined estradiol and trenbolone acetate (ETBA) implant containing 120 mg TBA and 24 mg E2 (*n* = 13, Revalor-S, Merck Animal Health), or (4) no implant (CON; *n* = 13). Different letters indicate a difference (*p* ≤ 0.05) between time points. Contrasts were used to compare differences in gain to feed over time between treatment and the control. The *p*-values for this analysis are displayed on the figure. The * indicates a difference (*p* < 0.05) between that treatment and the CON steers when analyzed as a repeated measure over time. All data are reported as LSMEANS ± SEM.

There were no differences (*p* ≥ 0.51) in initial steer weights at the start of the trial between treatments (Figure 1). When analyzed with repeated measures, weights of the steers increased (*p* < 0.0001) over time. Over the course of the trial, ETBA and TBA steers were heavier (*p* ≤ 0.02) than CON steers, while there was no difference in weight (*p* = 0.12) between E2 steers and CON steers. Similar results were observed when evaluating total gain and overall ADG (Table 2). There was no difference (*p* = 0.22) between E2 and CON steers in total gain and overall ADG. However, both ETBA and TBA steers had increased (*p* ≤ 0.04) overall ADG and total gain compared to CON steers, resulting in 25 and 13.4% improvements in overall ADG for ETBA and TBA steers, respectively. When DMI data was evaluated with repeated measures, DMI increased (*p* < 0.0001) over time (Figure 2). While on trial, ETBA steers had a 7% greater (*p* = 0.0003) DMI and TBA tended to have a 3.7% greater (*p* = 0.08) DMI than CON steers. No difference (*p* = 0.74) in DMI was observed between CON and E2 steers over the course of the trial. When G:F was analyzed over time, it was found that as time went on, G:F improved (Figure 3; *p* < 0.0001). Gain: feed was not different (*p* ≥ 0.30) between TBA, E2, and CON steers when analyzed as repeated measures. However, ETBA steers improved (*p* = 0.03) G:F by 14% when compared to CON steers.

### 3.2. Feeding Behavior 

**Figure 4:**
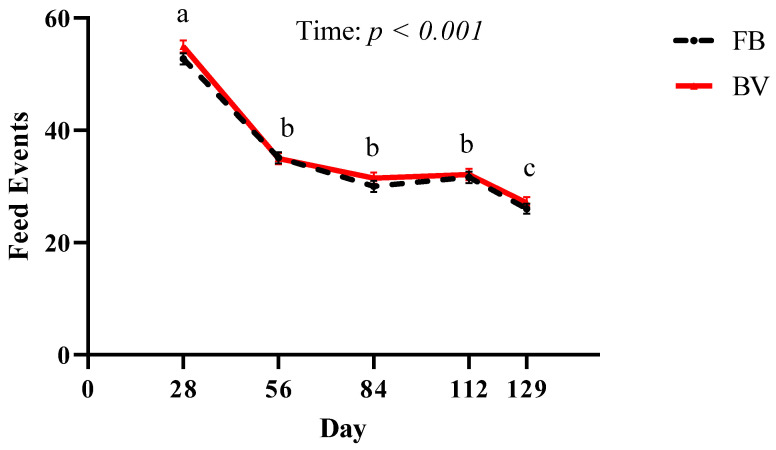
Feed events, feed bout (FB) and bunk visit (BV)**,** were measured as described in the Materials and Methods. Feed events were influenced (*p* ≤ 0.05) by time when analyzed as a repeated measure. Different letters indicate a difference (*p* ≤ 0.05) between time points. The *p*-value for this analysis are displayed on the figure. All data are reported as LSMEANS ± SEM.

**Figure 5:**
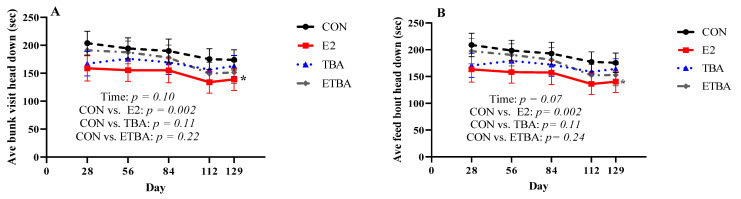

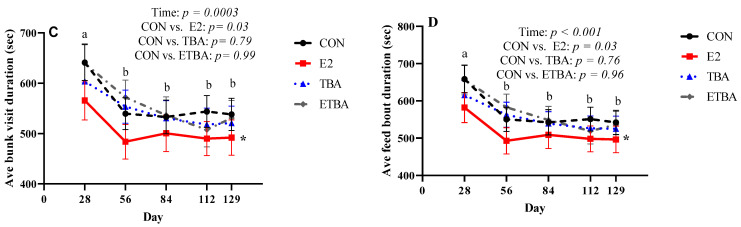
Meal events were measured as described in the Materials and Methods. Steers were initially stratified by weight and assigned to one of four treatments: (1) estradiol (E2) only implant containing 25.7 mg E2 (*n* = 12; Compudose, Elanco Animal Health), (2) trenbolone acetate (TBA) only implant containing 200 mg TBA (*n* = 12; Finaplix-H, Merck Animal Health), (3) a combined estradiol and trenbolone acetate (ETBA) implant containing 120 mg TBA and 24 mg E2 (*n* = 13, Revalor-S, Merck Animal Health), or (4) no implant (CON; *n* = 13). (**A**) average bunk visit head down, (**B**), average feed bout head down, (**C**) average bunk visit duration, and (**D**) average feed bout duration. Different letters, a, b, c, indicate a difference (*p* ≤ 0.05) between time points. Contrasts were used to compare differences in meal events over time between treatment and the control. The *p*-values for this analysis are displayed on the figure. The * indicates a difference (*p* < 0.05) between that treatment and the CON steers when analyzed as a repeated measure over time. All data are reported as LSMEANS ± SEM.

The effects of different anabolic implants in steers on feeding behavior was analyzed. Implants had no effect (*p* = 0.13) on FB or BV when compared to CON steers. However, as time went on, steers had fewer (*p* < 0.001) BV and FB (Figure 4), regardless of treatment. When meal events were investigated (Figure 5), it was found that E2 steers spent less time (*p =* 0.002) with their heads down per BV (Figure 5A) and FB (Figure 5B) than CON steers. There was no difference (*p =* 0.11) between CON and ETBA or TBA steers with time spent with their heads down per BV and FB. Estradiol steers also spent less time (*p* = 0.03) per each BV (Figure 5C) and FB (Figure 5D) compared to CON steers, while there was no difference (*p* = 0.79) between CON and TBA or ETBA steers with amount time spent per each FB and BV. 

### 3.3. Plasma and Liver Trace Mineral Concentrations

**Figure 6:**
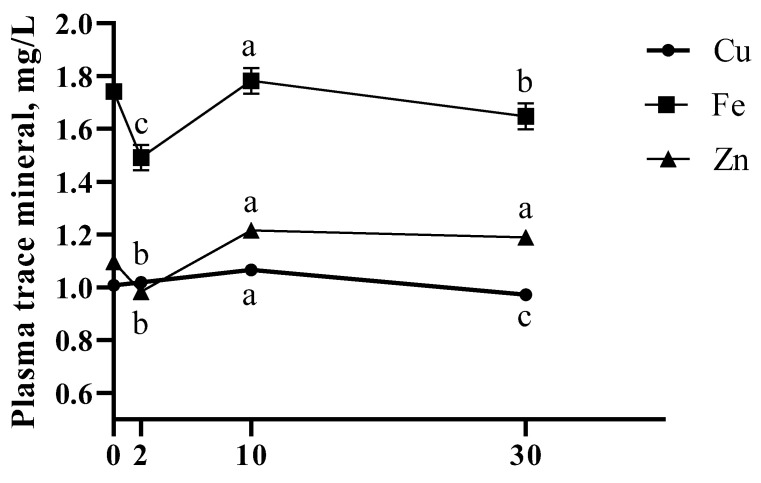
Plasma trace mineral concentrations were influenced throughout the implant trial. Plasma samples were collected on days 0, 2, 10, and 30. Day 0 values were utilized as a covariate in a repeated measures analysis and is displayed on the graph as a point of reference for initial plasma concentrations. Different letters indicate differences between (*p* ≤ 0.05) time points for each trace mineral analyzed. All data are presented as LSMEANS ± SEM. No Treatment × Time effects were observed for all trace minerals tested (*p* ≥ 0.18). Plasma Cu concentrations peaked on day 10 and were lesser than initial concentrations on day 30 (Time; *p* < 0.0001). Plasma Fe concentrations decreased on day 2 but increased by day 10 before decreasing again by day 30 (Time; *p* < 0.0001). Plasma Zn concentrations were lowest on day 2 before peaking on day 10 and remained steady through day 30 (Time; *p* < 0.0001).

The effects of different anabolic implants on plasma trace mineral concentrations was evaluated. Both E2 and TBA were not different (Table 3; *p* ≥ 0.13) from CON for plasma Cu, Fe, and Zn. However, ETBA plasma Zn was lesser than CON (*p* = 0.01), while not different for Cu and Fe (*p* ≥ 0.60). No effects of Treatment × Time (*p* ≥ 0.18) were observed for plasma measures. Time affected plasma Cu such that plasma Cu increased (*p* < 0.0001) from day 2 to 10 before decreasing below day 2 concentrations on day 30 (Figure 6). Additionally, both plasma Fe and Zn appear to decrease (*p* ≤ 0.0001) by day 2 followed by a sharp increase (*p* ≤ 0.0001) in plasma Fe and Zn concentrations on day 10. Plasma Fe slightly decreased (*p* = 0.02) by day 30 while plasma Zn remained constant (*p* = 0.28) through day 30 (Figure 6). Two days post-implant, liver Cu and Zn were lesser (*p* ≤ 0.04) for TBA than CON, while liver Mn tended (*p* = 0.06) to be lesser for TBA than CON (Table 4). Additionally, day 2 liver Zn concentrations were lesser for ETBA than CON (*p* = 0.04). By day 10, liver Cu and Zn concentrations were no longer different between TBA and CON (*p* ≥ 0.11), though TBA day 10 liver Mn remained lesser than CON (*p* = 0.0003). Day 10 liver Fe tended (*p* = 0.07) to be greater for E2 and was greater (*p* = 0.04) for ETBA than CON. No further effects (*p* ≥ 0.13) of E2, TBA, or ETBA vs. CON were observed for day 2 or 10 liver trace mineral concentrations. 

### 3.4. Serum Urea Nitrogen

Serum urea nitrogen was measured on days 0, 2, 10, 28, and 56 of the trial. When analyzed as repeated measures, anabolic implants, E2, TBA, or ETBA, had no effects (*p* ≥ 0.50) on SUN concentrations through day 56 of the trial when compared to the CON steers, as such the data was then analyzed investigating individual time-points, and once again anabolic implants had no effect (*p* ≥ 0.50) on SUN concentrations (Table 5). However, concentrations of SUN in the steers increased (*p* < 0.0001) over time (Table 5). 

### 3.5. Carcass Characteristics

Implant treatments had no effect (*p* ≥ 0.16) on dressing percentage, 12th rib fat thickness, or marbling when compared to CON steers (Table 6). Hot carcass weight was increased (*p* = 0.008) by 8% in ETBA steers compared to CON steers. The TBA steers had the largest REA compared to CON steers (*p* = 0.006), with it being increased by 10.7% (*p* *=* 0.006). Additionally, there was a trend for cold camera yield grade to be improved (*p* = 0.06) in TBA steers compared to CON steers. No further differences between implant treatments and CON were observed for HCW, REA, or cold camera yield grade (*p* ≥ 0.16).

### 3.6. Carcass Adjusted Growth 

When carcass-adjusted growth was evaluated, there were no differences (*p* ≥ 0.44) in carcass-adjusted final BW, carcass-adjusted total gain, carcass-adjusted ADG, or carcass-adjusted G:F between E2 and CON steers (Table 7) were found. However, ETBA (*p* = 0.01) had greater carcass-adjusted final BW than CON steers, though TBA (*p* = 0.24) was not different from CON. Additionally, carcass-adjusted total gain, ADG, and G:F were increased (*p* = 0.001) in ETBA steers compared to CON steers, while carcass-adjusted total gain and ADG tended to increase (*p* ≤ 0.10) in TBA steers compared to CON.

## 4. Discussion

In the U.S. over 90% of cattle receive at least one anabolic implant at some point during production [8], as anabolic implants increase overall performance and efficiency of beef cattle [4]. Implants have the added benefit of also increasing both the environmental sustainability of the industry [2] and economic return to producers [15]. However, despite the clear benefits of implanting, the exact physiological and molecular mechanism that anabolic implants operate through to increase overall growth and efficiency remains elusive [9,12]. Furthermore, when trace minerals are supplemented at higher concentrations recommended by feedlot consultants, rather than national research council recommendations, an increase in growth is observed [13]. This increase in growth is even further exacerbated when the animals receive anabolic implants, demonstrating that increased mineral concentrations may be required to support increased growth rates [13]. Therefore, the purpose of this research was to examine varying hormone type and concentration, estradiol only, trenbolone acetate only, or a combined estradiol and trenbolone acetate implant, on performance in the feedlot, feeding behavior, and concentrations of trace minerals in the plasma and liver of Angus sired steers, to help improve our understanding of anabolic implants. The brief findings of this study were that anabolic implants containing TBA improved growth, and altered trace mineral concentrations, while an E2 only implant altered steer feeding behavior. 

Current research suggests anabolic implants decrease land usage by 7.8–9.1% [2,19], and greenhouse gas emissions by 5.1% to 8.9% [2,19], creating a more environmentally sustainable end-product. This is through increasing ADG and G:F [4]. In a review published by Duckett and Pratt, the authors state that anabolic implants increase ADG by 18%, feed efficiency by 6%, and feed intake by 6% [4]. We found that a single anabolic implant containing 120 mg TBA and 24 mg E2 increased overall ADG by 25%, G:F by 14% and DMI by 7%. More recent research completed examining various implant protocols has found that anabolic implants can increase DMI from 5% to 12% [20]. One likely reason steers used in this trial had increased performance compared to the numbers reported by the review, is that there are variable responses to implants when used in different stages of production [21]. Specifically, cattle need adequate nutrition before implants can positively influence G:F and gain [21]. Additionally, the number of implants and type of implants given [20], the breed of cattle [15], and sex of cattle [22] can all influence how cattle respond to anabolic implants. The Duckett and Pratt review published an average of several studies [4], taken together with the multitude of factors influencing response to anabolic implants, this could explain the increase in performance that was observed in this trial. 

Interestingly in our trial, E2 steers did not have altered performance compared to CON steers. In a compilation of implant trials published, animals receiving either a single mild estrogen (around 20 mg estrogen) implant or a single strong estrogen implant (around 200 mg estrogen) had increased ADG and DMI compared to steers that never received an anabolic implant [23]. The payout period of anabolic implants is the effective period of the implant, which typically varies from 90–120 days [24], with the payout period being impacted by the hormone concentration [24]. The steers in the E2 group were implanted with Compudose, an implant containing 25.7 mg estradiol (Elanco Animal Health) with a 200 day payout, and according to the manufacturer, the payout occurs equally over the 200 days. The steers in this trial were harvested at 129 days, which may be part of the reason why E2 did not improve performance of the steers compared to CON.

Red Angus heifers categorized as having high ADG have longer FB durations than those heifers with a low ADG [17]. We have previously found that Angus sired steers have numerically greater feedlot performance and tended to have longer feed bouts and longer bunk visits than Santa Gertrudis sired steers [15]. In the current study, steers in the E2 group had shorter FB and BV, and spent less time with their heads down per BV and FB than CON steers. This is interesting, as there was no difference in performance between the E2 and CON steers. This suggests feeding behavior is not always related to feedlot performance, although more research needs to be done to determine the impacts of anabolic implants on feeding behavior.

Although the relationship between trace mineral nutrition and anabolic implants is not well understood, trace minerals can be linked to many aspects of growth. A clear connection between Zn and skeletal muscle protein synthesis has been observed using rodents to assess growth in response to Zn and protein supplementation [25]. Zinc is vital to cellular proliferation [26] and is a cofactor to metalloproteinases 2 and 9 [27], both of which are associated with increased proliferation rates [11], and protein turnover [10] in bovine satellite cells. Satellite cells are essentially muscle precursor cells [28] and are required to support skeletal muscle hypertrophy. Increasing satellite cell numbers allows for an increased capacity for skeletal muscle growth to occur [29]. Furthermore, the Cu dependent enzyme, lysyl oxidase, is responsible for maintaining the structural integrity of the extracellular matrix [30], a key component to proper muscle development. Given the strong molecular relationship between trace minerals and pathways associated with skeletal muscle growth, it is important that research is conducted to determine how different anabolic implants impact serum and liver concentrations of trace minerals.

Interestingly, both day 2 liver Cu and Zn were lesser for TBA than CON while day 2 liver Zn was lesser for ETBA than CON, suggesting the androgenic component of these treatments is influencing liver Cu and Zn more so than the estrogenic component. We have previously observed a decrease in liver Cu concentrations of implanted steers 14 days after a combination implant was administered, while liver Zn was greater for implanted steers than non-implanted at harvest [13]. In agreement with the current work, a decrease in liver Zn concentrations 14 days post-implant administration was observed, coinciding with a decrease in plasma Zn concentrations of implanted steers compared to non-implanted controls on day 13 that remained through day 73 [31]. The current study design was imperative to finding these TBA driven effects on liver Cu and Zn concentrations, as Niedermayer et al. [13] and Messersmith [30] both utilized combination implants that limited data interpretation to the effects of anabolic implant use rather than hormone type. Together, these data indicate trace mineral concentrations are influenced by hormone administration and hormone type. Additionally, it appears that trace minerals such as Cu and Zn, known for roles within many growth processes may be in greater demand by implanted cattle. 

Peak hormonal payout of implants has been observed within the first 40 days post-implant administration [24], indicating this time period should experience the greatest growth rates and subsequently, the greatest need for trace minerals to accommodate that growth. In the present study, the greatest differences in growth occurred within the first 28 days of implanting, interestingly, coinciding with many changes in trace mineral concentrations immediately following implant administration. These data emphasize the importance of trace mineral nutrition, especially Zn, during periods of high growth rates. Although liver Zn was lesser for TBA and ETBA than CON and liver Cu lesser for TBA than CON on day 2, no differences were observed by day 10. However, Niedermayer et al. [13] and Messersmith [31] still observed differences in liver trace mineral concentrations 14 days post administration of a combination implant. This difference may be due to the implant potencies used. Both previously mentioned studies [13,31] utilized aggressive combination implants (Component TE-200; 200 mg trenbolone acetate and 20 mg estradiol; Elanco Animal Health) compared to the less aggressive estrogen or trenbolone acetate only or combination implant (Revalor-S; 120 mg trenbolone acetate and 24 mg estradiol; Merck Animal Health) used in the current study. 

Indeed, implant hormone potency and type can influence mineral stores. The observed increase in day 10 liver Fe concentrations for E2 and ETBA treatments compared to CON indicates a role for E2 in Fe metabolism. Research has found that E2 impairs the transcription of the Fe exporter, ferroportin, through an E2 responsive element [32]. Therefore, steers implanted with E2 appeared to have limited Fe export from the liver in the current study, however, no effects of E2 implant strategies were observed for plasma Fe concentrations. In addition to Fe metabolism, emerging research has found that heifers implanted with an aggressive two implant strategy (Revalor-200, Merck Animal Health; on days 0 and 91) had lesser liver Mn concentrations than heifers implanted with an extended-release implant (Revalor-XH, Merck Animal Health) on day 0 [33]. Interestingly, both Niedermayer et al. [13] and Messersmith [31] observed decreases in liver Mn concentrations of aggressively implanted steers 14 days post implant administration. These data are in agreement with the decrease observed in liver Mn of TBA steers compared to CON on day 2 and 10. However, the lack of differences in liver Mn due to ETBA suggests either the lesser concentration of TBA in the ETBA implant did not as aggressively affect skeletal muscle protein degradation as the TBA implant alone, or that the addition of E2 in the combination implant supported more skeletal muscle net protein gain. Regardless, the decrease in liver Mn may be due to less skeletal muscle protein degradation in implanted cattle resulting in lesser demand for the urea cycle. Therefore, the Mn dependent terminal enzyme of the urea cycle, arginase [34,35], is likely down regulated leading to the decrease in liver Mn observed. However, more work is warranted to confirm how liver Mn is being utilized.

Serum urea nitrogen is a marker of lean tissue anabolism, as it inversely indicates increased N retention [7]. Implant strategies have been shown to impact SUN concentrations [20], as the hormones used in implants increase protein accumulation in vivo [36] and protein synthesis rates in vitro [10]. In the present study, SUN was investigated on days 0, 2, 10, 28 and 56 from CON, E2, TBA, and ETBA steers. Interestingly, no differences were observed in SUN for these different implant treatments. Research conducted using a different combination estradiol trenbolone acetate implant did not find a difference in SUN until day 213 [20]. Another study that investigated the effects of anabolic implants on plasma urea nitrogen (PUN) found that steers that received a mild implant (14 mg E2 and 80 mg TBA) and were re-implanted 56 days later with a more aggressive implant (20 mg E2 and 200 mg TBA) had decreased PUN on day 70 of the trial [13]. These findings taken together suggest that the implant protocol and strength of the implant influence SUN concentrations in steers. 

As the use of anabolic implants increases, so does the concern with quality grade of the beef [37,38], which is a key component of the grid system used to determine payments to producers for producers in the United States [39]. A combined high quality grade and low yield grade is optimal for producers paid on the grid system [39]. Increased marbling increases quality grade, while increased subcutaneous fat undesirably increases yield grade [40]. Estradiol and TBA have been shown to decrease both marbling [6] and subcutaneous fat [37]. In the current study, none of the implant treatments altered marbling, while TBA steers only tended to have improved yield grade compared to CON steers. This is most likely explained as the steers were finished at a group average of 7 mm of rib fat and implants were administered 129 days prior to harvest, both of which helped to minimize any negative effects of implants on carcass characteristics. Research has found that giving cattle implants earlier in the feeding period, rather than later, helps offset potential negative effects of implants on marbling [41]. Additionally, as previously mentioned, peak payout of the implants typically occurs within the first 40 days post-implanting with most anabolic implants having an effective payout period of 90–120 days [24]. As the steers were finished harvest at day 129 post-implanting, the negative effects that anabolic implants sometimes have on marbling were not observed in this trial. If the steers were finished to a set weight or were kept on feed longer to reach the U.S. industry standard of 12 mm of rib fat, differences may have been observed in both marbling and yield grade. 

Steers in the ETBA group did have increased HCW when compared to the CON steers, with the ETBA implant increasing HCW by 8%. In Duckett and Pratt’s review, they found that on average implants increased carcass weight by 5% [4]. Interestingly, TBA implants increased REA by 10.7% compared to CON steers, most likely due to increased skeletal muscle protein accretion and muscle growth. Additional emerging research has found that increasing the hormone concentration of anabolic implants leads to an increase in HCW and REA linearly in yearling beef steers [42]. These findings, taken together, help confirm that anabolic implants increase HCW and REA of cattle. 

## 5. Conclusions

In summary, the present study found that a single moderate potency ETBA (124 mg TBA and 24 mg E2) implant improves ADG by 25%, G:F by 14%, and HCW by 8% compared to non-implanted steers. In steers that receive only a TBA (200 mg) implant, REA is increased by 10.7%. However, a single E2 implant did not impact performance when compared to CON steers. The information gained in this trial adds to the body of knowledge confirming that implants containing TBA are an effective tool to increase overall growth and efficiency of cattle and showcases differences in feedlot performance, feeding behavior, and carcass quality when animals are administered implants with different hormones and/or concentrations. Additionally, these data indicate hormone content of anabolic implants influences liver and plasma trace mineral concentrations. Specifically, provision of a TBA only implant has effects on Zn and Mn liver concentrations. These data suggest skeletal muscle protein synthesis and degradation are influenced by administration of a TBA only implant. However, future work is needed to help decipher the physiological and molecular mechanisms that anabolic implants operate through to increase skeletal muscle growth and efficiency in cattle, as well as improving the understanding of the relationships between trace minerals and anabolic implant stimulated skeletal muscle growth. 

## Figures and Tables

**Table 1:** Composition and nutritional analysis of background and finishing diets fed to Angus steers throughout the trial. ^1^

-	Background Diet, (%)	Finishing Diet, (%)
**Feed (% DM)**	-	-
Corn Silage	17.9	-
Haylage	17.9	15.38
High Moisture Corn	17.2	40.00
Cracked Barley	17.2	35.58
Alfalfa	28.7	7.69
Background Mineral ^2^	0.89	-
Finishing Mineral ^3^	-	1.53
**Analysis (% DM)**		
Moisture	35.4	22.0
Crude Protein	15.4	13.0
ADF	22.6	14.0
NDF	29.7	21.6
Net Energy_(m)_^4^	1.72	1.96
Net Energy_(g)_^4^	1.10	1.32
Minerals (DM)	-	-
Calcium (%)	1.09	0.68
Phosphorus (%)	0.37	0.34
Manganese (mg/kg)	101	126
Zinc (mg/kg)	104	137
Copper (mg/kg)	31	31

^1^ Background diet was fed day 0–10 of trial, steers then received a series of step-up diets incrementally increasing percent concentrate (DM basis) until the finishing ration was reached. Finishing ration was fed day 41–129. ^2^ Composition of background mineral (DM basis): 12.43% Ca, 8.13% Cl, 0.52% Mg, 8.29% P, 0.52% K, 4.87% Na, 0.81% S, 10.36 mg/kg Co, 2071 mg/kg Cu, 4143 mg/kg Fe, 4972 mg/kg Mn, 26.9 mg/kg Se, 6215 mg/kg Zn, and 1825 mg/kg Monensin. ^3^ Composition of finishing mineral (DM basis): 16.73% Ca, 11.09% Cl, 0.23% Mg, 0.31% P, 0.52% K, 6.73% Na, 0.32% S, 10.46 mg/kg Co, 941 mg/kg Cu, 2614 mg/kg Fe, 5018 mg/kg Mn, 10.46 mg/kg Se, 6273 mg/kg Zn, and 921 mg/kg Monensin. ^4^ Net energy for maintenance (_m_) and gain (_g_) are presented as Mcal/kg.

**Table 2:** Average daily gain throughout the feedlot period of Angus steers receiving different implant strategies.

		Implant Treatments ^1^				*p*-Values of Contrasts ^2^		
	CON	E2	TBA	ETBA	SEM	E2 vs. CON	TBA vs. CON	ETBA vs. CON
Steers (*n*)	**13**	**11**	**12**	**11**	-	-	-	-
Average Daily Gain (kg)								
Day 0–28	0.93	1.32	1.41	1.46	0.12	0.03	0.007	0.003
Day 28–56	1.34	1.39	1.32	1.69	0.18	0.81	0.93	0.05
Day 56–84	2.44	2.07	2.50	2.20	0.18	0.13	0.78	0.30
Day 84–112	1.21	1.53	1.49	1.80	0.20	0.17	0.22	0.01
Day 112–129	1.70	1.87	2.00	2.24	0.27	0.66	0.39	0.14
Day 0–129	1.49	1.61	1.69	1.88	0.07	0.20	0.03	0.0002
Total Gain (kg)	193.07	208.18	217.87	241.83	8.80	0.22	0.04	0.0002

^1^ Implant treatments administered on day 0 include: no implant (CON), Compudose (E2; 25.7 mg estradiol), Finaplix-H (TBA; 200 mg trenbolone acetate), and Revalor-S (ETBA; 120 mg trenbolone acetate + 24 mg estradiol). ^2^ Contrast statements were formed to test differences between E2, TBA, or ETBA vs. CON treatment.

**Table 3:** Plasma trace mineral concentrations of Angus steers receiving different implant strategies.

	Implant Treatments ^1^					*p*-Values of Contrasts ^2^		
	CON	E2	TBA	ETBA	SEM	E2 vs. CON	TBA vs. CON	ETBA vs. CON
Steers (*n*)	**12**	**12**	**12**	**12**				
Plasma ^3^, mg/L								
Cu	1.01	1.01	1.04	1.03	0.027	0.99	0.34	0.60
Fe	1.65	1.60	1.64	1.68	0.089	0.57	0.91	0.73
Zn	1.17	1.16	1.12	1.07	0.029	0.71	0.13	0.01

^1^ Implant treatments administered on day 0 include: no implant (CON), Compudose (E2; 25.7 mg estradiol), Finaplix-H (TBA; 200 mg trenbolone acetate), and Revalor-S (ETBA; 120 mg trenbolone acetate + 24 mg estradiol). ^2^ Contrast statements were formed to test differences between E2, TBA, and ETBA vs. CON treatment. ^3^ Data were analyzed as repeated measures with the repeated effect of time. Plasma was collected on day 0, 2, 10, and 30. Day 0 values were utilized as covariates in analysis. No Treatment × Time effects were observed (*p* ≥ 0.18).

**Table 4:** Liver trace mineral concentrations following implant administration of Angus steers receiving different implant strategies.

	Implant Treatments ^1^					*p*-Values of Contrasts ^2^		
	CON	E2	TBA	ETBA	SEM	E2 vs. CON	TBA vs. CON	ETBA vs. CON
Steers (*n*)	**12**	**12**	**12**	**12**				
Liver, mg/kg DM								
Day 2								
Cu	309	273	233	255	26.1	0.31	0.03	0.13
Fe	307	307	277	287	24.5	0.99	0.39	0.57
Mn	9.0	8.5	7.7	8.1	0.50	0.46	0.06	0.19
Zn	111	109	93	93	6.4	0.77	0.04	0.04
Day 10								
Cu	333	335	284	319	25.5	0.97	0.18	0.69
Fe	241	289	261	296	22.9	0.07	0.45	0.04
Mn	9.4	8.8	7.3	8.9	0.41	0.26	0.0003	0.29
Zn	119	115	104	124	8.1	0.66	0.11	0.60

^1^ Implant treatments administered on day 0 include: no implant (CON), Compudose (E2; 25.7 mg estradiol; Elanco Animal Health), Finaplix-H (TBA; 200 mg trenbolone acetate; Merck Animal Health), and Revalor-S (ETBA; 120 mg trenbolone acetate + 24 mg estradiol; Merck Animal Health). ^2^ Contrast statements were formed to test differences between E2, TBA, and ETBA vs. CON treatment.

**Table 5:** Serum urea nitrogen concentration of Angus steers receiving different implant strategies.

		Implant Treatments ^1^					*p*-Values of Vontrasts ^2^		
	CON	E2	TBA	ETBA	All Implants ^3^	SEM	E2 vs. CON	TBA vs. CON	ETBA vs. CON
Steers (*n*)	13	12	12	13	50				
Serum Urea Nitrogen (mg/dL)									
Day 2	7.08	7.46	9.30	8.52	8.26 ^a^	1.36	0.84	0.23	0.40
Day 10	9.43	9.43	9.77	9.83	9.69 ^a^	1.05	0.99	0.80	0.76
Day 28	13.19	13.24	11.79	12.99	12.77 ^b^	0.87	0.97	0.24	0.87
Day 56	11.42	12.47	10.12	10.81	11.24 ^b^	1.22	0.53	0.43	0.70

^1^ Implant treatments administered on day 0 include: no implant (CON), Compudose (E2; 25.7 mg estradiol), Finaplix-H (TBA; 200 mg trenbolone acetate), and Revalor-S (ETBA; 120 mg trenbolone acetate + 24 mg estradiol). ^2^ Contrast statements were formed to test differences between E2, TBA, and ETBA vs. CON treatment. ^3^ Analysis with repeated measures determined serum urea nitrogen concentrations were affected by time (*p* < 0.0001). Differences (*p* < 0.05) between time points are indicated by different superscript letters.

**Table 6:** Carcass characteristics of Angus steers receiving different implant strategies.

		Implant Treatments ^1^				*p* Values of Contrasts ^2^		
	CON	E2	TBA	ETBA	SEM	E2 vs. CON	TBA vs. CON	ETBA vs. CON
Steers (*n*)	13	11	12	11				
Dressing Percentage	60.1	59.3	60.4	60.2	0.46	0.21	0.61	0.86
Hot Carcass Weight (kg)	317	311	330	343	7.41	0.57	0.16	0.008
Marbling Score ^3^	486	468	483	423	36	0.71	0.94	0.16
Ribeye Area (cm^2^)	70.71	72.65	78.06	74.06	2.13	0.51	0.006	0.22
12th Rib Fat Thickness (mm)	7.67	7.65	7.49	7.85	0.23	0.91	0.41	0.59
Cold Camera Yield Grade	2.99	2.91	2.65	3.08	0.16	0.67	0.06	0.67

^1^ Implant treatments administered on day 0 include: no implant (CON), Compudose (E2; 25.7 mg estradiol), Finaplix-H (TBA; 200 mg trenbolone acetate), and Revalor-S (ETBA; 120 mg trenbolone acetate + 24 mg estradiol). ^2^ Contrast statements were formed to test differences between E2, TBA, and ETBA vs. CON treatment. ^3^ 300 to 399 = slight, 400 to 499 = small, 500 to 599 = modest.

**Table 7:** Carcass adjusted performance of Angus steers receiving different implant strategies.

		Implant Treatments ^1^				*p*-Values of Contrasts ^2^		
	CON	E2	TBA	ETBA	SEM	E2 VS. CON	TBA vs. CON	ETBA vs. CON
Steers (*n*)	13	11	12	11				
Final BW ^3^ (kg)	527	526	546	570	11.69	0.96	0.24	0.01
Total Gain ^4^ (kg)	212	221	231	255	8.34	0.44	0.10	0.0006
ADG ^5^ (kg)	1.64	1.71	1.79	1.98	0.06	0.44	0.10	0.0006
G:F ^6^	0.14	0.15	0.15	0.16	0.004	0.30	0.16	0.0033

^1^ Implant treatments administered on day 0 include: no implant (CON), Compudose (E2; 25.7 mg estradiol), Finaplix-H (TBA; 200 mg trenbolone acetate), and Revalor-S (ETBA; 120 mg trenbolone acetate + 24 mg estradiol). ^2^ Contrast statements were formed to test differences between E2, TBA, and ETBA vs. CON treatment. ^3^ Carcass adjusted final body weight of the steers was calculated by dividing the individual animal’s hot carcass weight by the individual animal’s dressing percentage. ^4^ Carcass adjusted total gain was calculated by subtracting the initial body weight with a 4% shrink applied from the carcass adjusted final body weight. ^5^ Carcass adjusted average daily gain was calculated by taking the carcass adjusted gain and dividing by total days on feed (129). ^6^ Carcass adjusted gain to feed was calculated by dividing total carcass adjusted gain by total dry matter intake.

## Data Availability

The data presented in this study are available upon request from the corresponding author. The data are not publicly available due to restrictions in place by the funding agency.
